# The medical pause: Importance, processes and training

**DOI:** 10.1111/medu.14529

**Published:** 2021-05-01

**Authors:** Joy Yeonjoo Lee, Adam Szulewski, John Q. Young, Jeroen Donkers, Halszka Jarodzka, Jeroen J. G. van Merriënboer

**Affiliations:** ^1^ School of Health Professions Education Maastricht University Maastricht The Netherlands; ^2^ Departments of Emergency Medicine and Psychology Queen’s University Kingston ON Canada; ^3^ Department of Psychiatry Donald and Barbara Zucker School of Medicine at Hofstra/Northwell and the Zucker Hillside Hospital at Northwell Health Glen Oaks NY USA; ^4^ Faculty of Education Sciences Open University Heerlen The Netherlands

## Abstract

Research has shown that taking ‘timeouts’ in medical practice improves performance and patient safety. However, the benefits of taking timeouts, or pausing, are not sufficiently acknowledged in workplaces and training programmes. To promote this acknowledgement, we suggest a systematic conceptualisation of the medical pause, focusing on its importance, processes and implementation in training programmes. By employing insights from educational and cognitive psychology, we first identified pausing as an important skill to interrupt negative momentum and bolster learning. Subsequently, we categorised constituent cognitive processes for pausing skills into two phases: the decision‐making phase (determining when and how to take pauses) and the executive phase (applying relaxation or reflection during pauses). We present a model that describes how relaxation and reflection during pauses can optimise cognitive load in performance. Several strategies to implement pause training in medical curricula are proposed: intertwining pause training with training of primary skills, providing second‐order scaffolding through shared control and employing auxiliary tools such as computer‐based simulations with a pause function.

## Abstract 2

Lee et al. suggest a systematic conceptualization of the medical pause, focusing on its importance, processes, and implementation in training programs.

## 1 INTRODUCTION

Research has revealed that medical errors are among the leading causes of preventable deaths each year.^1^, ^2^ The widespread agreement that health care delivery can harm patients has spurred a movement towards quality improvement and diverse strategy application to improve patient safety.^3^ The *timeout*, where the current course of action is paused and the parameters of safe care are reassessed, has been adopted as safety strategy in many settings.^4^, ^5^ Although studies have shown that taking timeouts significantly decreases patient morbidity and mortality,^6^, ^7^, ^8^ compliance with timeout protocols appears to be low, diluting its clinical benefit.^5^, ^9^, ^10^

We posit that the basic nature of the timeout is *pausing*, a conscious decision to stop current performance for a physical time that allows for additional cognitive activities. While medical timeouts in general refer to a formal practice with structured protocols in a team setting (eg surgical timeout, diagnostic timeout),^11^, ^12^ pausing can occur either individually or collectively, with or without formal protocols. Pausing as a general construct and strategy can potentially advance safety and learning in medicine. Yet, to date, we do not have a theoretical understanding of what constitutes a pause and how pausing might benefit clinical outcomes and spur cultural changes.^13^

In the following, we aim to explicate the importance, processes and training of the medical pause. First, we will focus on the unique advantages of pausing: intercepting negative momentum and promoting learning (Importance). Second, we will describe a new conceptual framework of pausing by analysing its constituent cognitive processes (Processes). Third, moving from a theoretical basis to practical implementation, we will suggest several strategies to train pausing within the medical curriculum (Training). Based on our combined experience in both educational psychology and clinical work, we will integrate existing theories from various fields (eg cognitive psychology and complex learning), resulting in a systematic conceptualisation of the medical pause.

## 2 IMPORTANCE: THE STRENGTHS OF PAUSING

**FIGURE 1:**
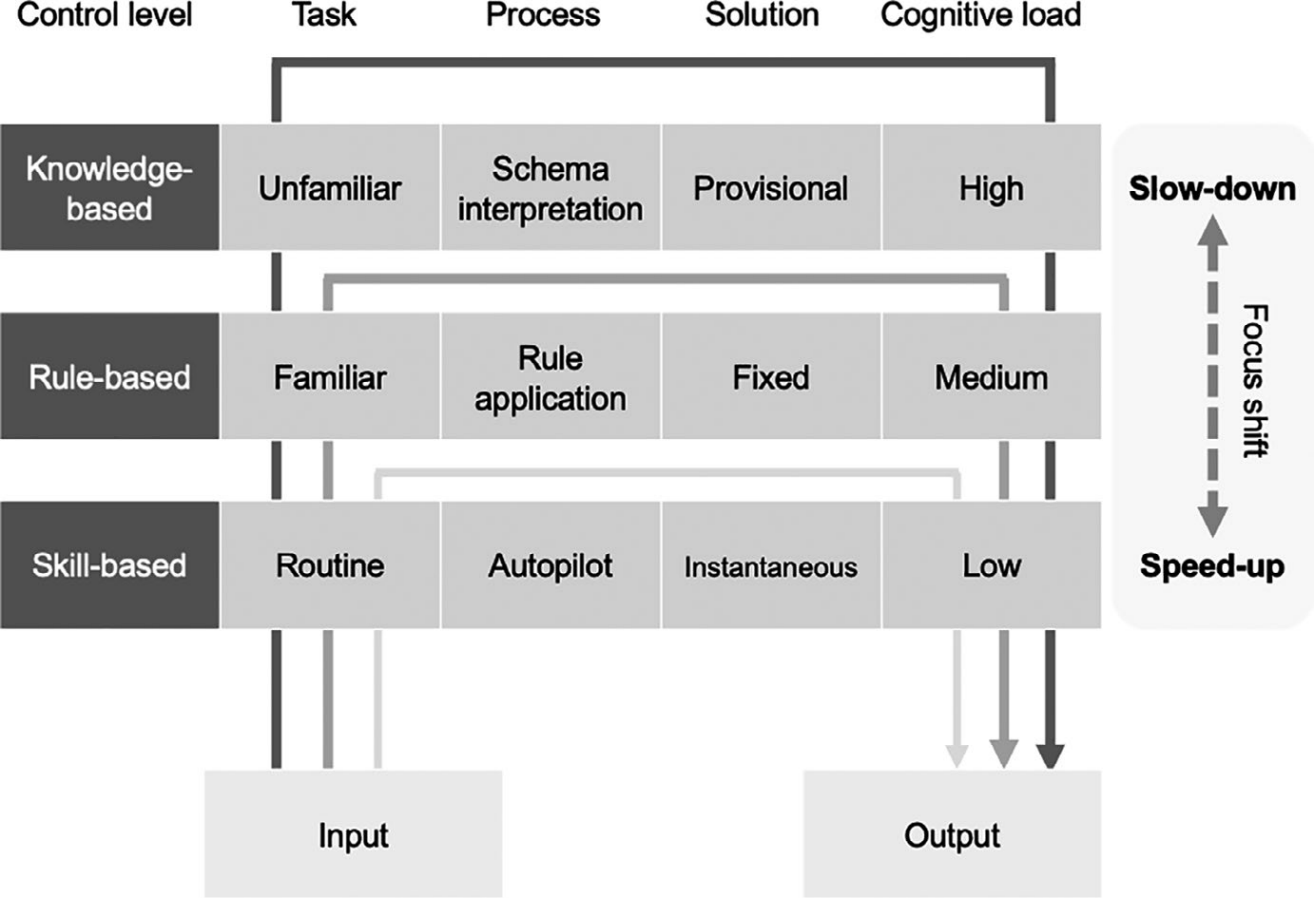
Three levels of cognitive control in real‐life problem solving (skill‐based, rule‐based, and knowledge‐based). The two‐way arrow on the right side represents two different directions in focus changing across these levels (slowing down and speeding up)

Problem solving is one of the major approaches to clinical reasoning.^14^ Using an elaborated version of dual‐process theories^15^, ^16^ and cognitive load theory,^17^ we will describe how clinical problem solving is guided by three different levels of cognitive control: skill‐based, rule‐based and knowledge‐based control (Figure 1).

*Skill‐based control* (SC) is activated when dealing with a routine task, using cognitive rules internalised through repetitive practice. When faced with a patient who has ventricular fibrillation, an experienced emergency physician can quickly ensure that the team prioritises chest compressions and defibrillation. With years of training and experience, this process becomes automatic and intuitive (ie autopilot). When problems are not entirely routine but familiar enough to apply internal or external rules, *rule‐based control* (RC) is initiated.^16^ Using checklists during surgical timeouts or following step‐by‐step instructions for procedures like central line insertion are examples of this process. For atypical and novel problems, *knowledge‐based control* (KC) is stimulated, where ‘knowledge’ means mental models or schemas of a domain that reflect how concepts in the domain are structured.^18^ When diagnosing a patient with a complex presentation (eg adrenal insufficiency as a cause of hypotension), a physician may need to consider alternative diagnoses by reflecting on similar cases and building upon relevant knowledge. Whereas the level of conscious control and its derived cognitive load are highest in the case of KC, they are lowest when practising SC.^19^

In real‐life problem solving, these three levels run in parallel, with weighted focus depending on the activity.^16^ Figure 1 depicts how this focus shifts across the levels: *slowing down* ^20^ as the focus moves from SC to KC, and *speeding up* when the opposite is true. During a routine operation, a surgeon who notices atypical anatomy would pause the procedure to reassess a list of alternatives and carefully apply the selected alternative (ie slowing down). Once this step is completed, this surgeon would return to the faster autopilot mode (ie speeding up). Note that this transition is feasible only for experienced performers since novices can neither identify cues that initiate the transition nor effectively change their focus as they lack structured schemas and skills.

While the speeding‐up process has been highlighted in the professional skill acquisition literature,^21^ the slowing‐down process has been emphasised in the context of safety culture and expertise maintenance in medicine.^22^ Moulton et al identified slowing down as a crucial component of medical expertise, coining the phrase ‘slowing down when you should’.^23^ They characterised pausing as the most extreme form of slowing down.^24^ While slowing down has been described as a cognitive process that may accompany physical manifestation, we further specify pausing as a cognitive and temporal process grounded in the physical world. This physicality grants pausing two unique advantages: pausing allows for (1) explicit interception of negative momentum and (2) dedicated time for necessary processing at the KC level, such as learning.

In experimental psychology, *negative momentum* is a state stimulated to move away from desired goals.^25^ Using an analogy from physical laws, the psychological state keeps its current motion, unless extra force is applied to change that state.^26^ We identified two examples of negative momentum in medicine: the *hurry‐up syndrome* ^27^ and *drifting*.^24^ The term hurry‐up syndrome has been used in aviation safety research which reveals that time pressure is significantly correlated with errors and safety risks.^27^ In medicine, time pressure and clinical acuity can cause the hurry‐up syndrome, resulting in medical errors and safety issues. Facing this negative momentum, novices tend to act quickly but inappropriately. To encourage them to rather stay undetermined and take a pause, experienced surgeons teach their residents the phrase, ‘Do not just do something, stand there’.^8^

While this undetermined neutral status can be beneficial sometimes, mind wandering or inattentiveness can be less advantageous, comprising another example of negative momentum.^28^ Contrary to the hurry‐up syndrome, drifting is the distracted state arising from ‘boring’ tasks and complacency.^24^ During routine tasks in an operation, surgeons could become careless and join extraneous chatting with colleagues, failing to monitor adverse events as they emerge.^24^ They inappropriately remain at the SC level, without allocating the cognitive resources that are freed up by automatic processing to essential activities.

After direct interruption of these types of negative momentum, pausing inserts a certain period of time that not only makes the interruption stronger but also facilitates additional processing at the KC level. We postulate that this processing promotes learning since it allows for a reconstruction of schemas. Ericsson^29^ argued that expert performance can be maintained by continuously seeking out learning opportunities, referred to as *deliberate practice*. Deliberate practice includes resisting the tendency towards complacency and deliberately reconstructing schemas by spending additional time analysing everyday performance.^29^ Since learning opportunities from pausing arise during performance, they are strongly fostered by fresh memories and embodied experiences that pre‐ or post‐timeouts cannot provide.

The benefits of pausing are evident and long‐term compared with the costs. Rall et al^8^ have shown that if trained sufficiently, only 10 seconds of timeout can be effective to improve performance in an emergency setting. The time lost taking timeouts is offset by improved team action, while the delay does not significantly jeopardise performance.^8^ Moreover, enhanced safety will not only prevent patients from suffering from complications but also protect health care professionals from suffering psychologically from medical errors.^30^

## 3 PROCESSES: KEY COMPONENTS OF PAUSING

We identified two phases of pausing: the decision‐making phase to determine when and how to take pauses and the executive phase to apply cognitive activities such as relaxation and reflection during the pauses. To make pausing skills effective, multiple cognitive and metacognitive processes must be functioning across these phases.

### 3.1 Decision‐making phase

While negative momentum should be interrupted, there is also *positive momentum* that should not be interrupted but rather supported during medical workflow; in other words, ‘not slowing down when you should not’. In extreme circumstances where expediency must be prioritised (eg emergency caesarean section), the opportunity to slow down does not exist. In team performance, taking an individual timeout could interrupt collective performance. Literature in psychology and brain sciences has confirmed that slowing down may impair performance depending on the situation.^31^, ^32^ Therefore, deciding on whether or not to pause becomes a highly advanced clinical judgement, involving time management to assess the time available and the risk of using the time.^8^

#### 3.1.1 Planning

The deployment of pauses can take two forms: proactive planning or responsive improvisation.^33^ Pausing can be proactively planned before a task, by identifying landmines that require special attention.^33^ These landmines are determined by assessing the task complexity and the performer's own capability. Through this assessment and mental simulation, one can establish a game plan^33^ where measures for the landmines are arranged. For instance, a surgeon who previously experienced difficulties performing lung resection might plan a pause before the challenging portion of the procedure. Expert performers are good game planners who excel in predicting what is going to happen during a procedure, thanks to their superior mental simulation.^33^

#### 3.1.2 Improvising

Responsively improvised pauses, on the other hand, take place when encountering unexpected events (eg recognising an abnormality in patients’ anatomy during surgery) or unsolvable problems (eg initial treatment not working during resuscitation). Realising unexpected landmines along the way, performers must make emergency decisions on taking a pause. This form of decision making is extra demanding because it involves the adjustment of the original game plan, quick problem solving, accurate calculation of the time available and constant vigilance for signals to pause.^33^

#### 3.1.3 Self‐monitoring

Although this vigilance is a prerequisite for pause deployment, the negative momentum is sometimes the result of vigilance decrement.^34^ In considering this dilemma, we argue that introducing metacognition,^35^ another level of cognitive processes that can oversee cognition itself, might be a solution. Self‐monitoring is a metacognitive process to supervise one's own thoughts and mental status.^18^ Overseeing one's own monitoring capability could be a measure to help prevent vigilance decrement.

#### 3.1.4 Cognitive load

We use cognitive load theory (CLT)^19^, ^36^ to further explain *what* and *how* to self‐monitor. CLT presumes that diverse mental processes evoke cognitive load on working memory and that task performance deteriorates if the load exceeds working memory capacity. Research has shown that vigilance produces a tangible amount of cognitive load,^34^, ^37^ which means performers should always reserve sufficient capacity for this process in working memory.^23^ To stress the importance of this reservation, we suggest a new typology of cognitive load: primary load (PL) caused by domain‐specific primary tasks (eg a surgical task), secondary load (SL) from domain‐general processes that support the primary tasks (eg self‐monitoring during surgery) and extraneous load (EL) that does not contribute to the tasks (eg distracting conversations during surgery). Thus, the targets of self‐monitoring are narrowed down to two constructs: whether the room for SL is reserved and whether the total load is below the limit.

Conceptualising cognitive load and its typology with explicit terminology guides performers on how to self‐monitor. It facilitates the conscious control of behaviour,^38^ providing a useful internal cue for self‐monitoring that can be measured by introspection.^39^ In various medical domains, cognitive load is known as a valid indicator of expertise, performance, and learning.^39^, ^40^, ^41^, ^42^ The internality of the cue is valuable in many domains of medicine, where access to information on decision support may be limited and professionals often work alone or with less experienced staff who may not be able to provide high‐quality feedback.^23^

#### 3.1.5 Collective decision making

In team performance, more external cues and interventions are necessary. To develop external cues, the team could use real‐time clinical decision support^43^ and encourage themselves to think aloud during performance. Based on this shared mental profile, the team leader or a separate overseer could make decisions to improvise pauses. When the cues are insufficient, forced pauses initiated by formal timeout protocols can be of benefit.^44^ I‐PASS^45^ and SBAR^46^ are good examples of organisational efforts to implement interventions to facilitate medical pauses.

### 3.2 Executive phase

Once a decision to pause is made, the processes to optimise internal and external resources ensue to redirect the negative momentum. In the stress literature, this optimisation is essential to titrate the stress level below distress while maintaining the eustress status, thereby maximising performance.^47^, ^48^ Similarly in CLT, to promote performance and learning, cognitive load should be kept below working memory capacity, but not too low in order to maintain PL and SL.

We identified two types of processes to find the optimal level: *relaxation* that reduces overload in working memory and *reflection* that interconnects working memory and long‐term memory to restructure schemas. In difficult resuscitation cases, a physician may pause the case to calm down (relaxation) and ‘recap’ the current situation by summarising previous performance, asking for others’ ideas, and preparing next priorities, based on experience and knowledge (reflection). Experienced physicians are reportedly better skilled in these coping strategies,^41^ likely because of their honed reasoning based on the assessment of internal and external resources in given situations.^49^

#### 3.2.1 Relaxation

**FIGURE 2:**
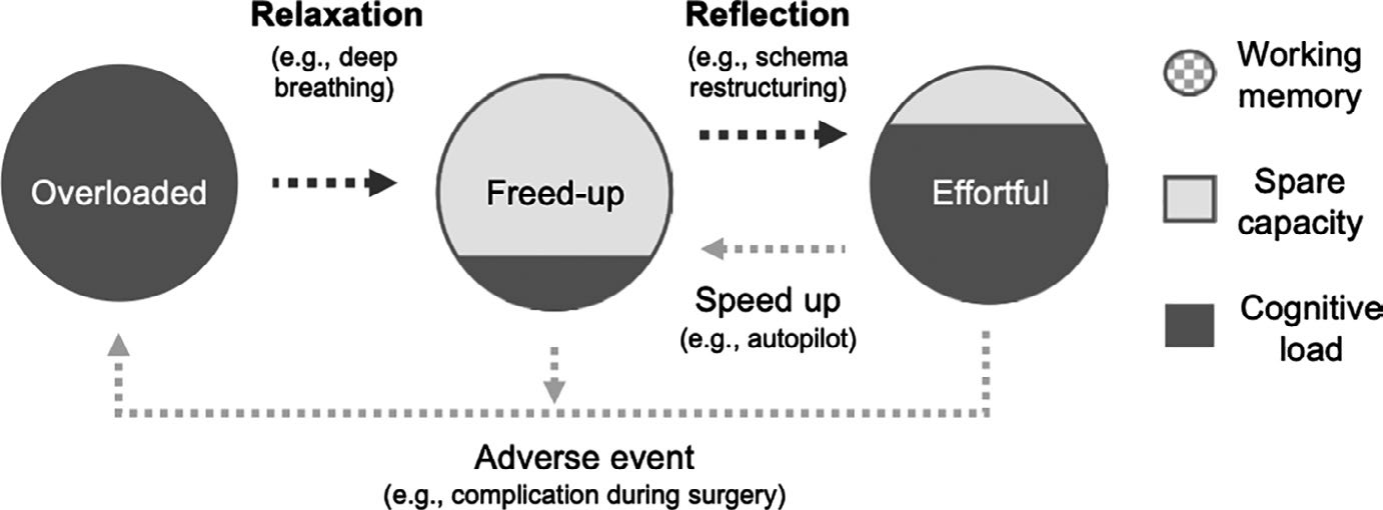
The cognitive load state transition triggered by relaxation and reflection during pauses, as well as speeding up and adverse events. Since some tasks do not allow for the opportunity to take a pause, deciding on whether or not to pause becomes a highly advanced clinical judgement

Figure 2 describes the state transition of the cognitive load level triggered by several factors as well as relaxation and reflection. The state of being *overloaded*, sometimes referred to as ‘helmet fire’, is caused by adverse events such as time pressure, life‐threatening complications, and overwhelmingly complex task environments. This results in acute stress and heightened emotions, contributing to either PL or EL depending on the task.^50^ Through relaxation processes, the overloaded state can transition to the *freed‐up* state, as the source of overload is managed or the depletion of working memory is recovered through cognitive rest.^51^

Among several relaxation techniques,^52^ deep breathing has been recommended for clinical settings as the fastest and simplest technique.^53^ Deep breathing, or diaphragmatic breathing, works as a brief form of meditation inserted within performance.^54^ Empirical studies show that it reduces stress, anxiety, self‐doubt and cortisol levels,^55^ also benefiting performance and learning, such as working memory enhancement, and motor skill acquisition and retention.^56^, ^57^

#### 3.2.2 Reflection

The freed‐up state is a transient phase where relaxation or the speeding‐up processes have created spare capacity for potentially any type of cognitive load to be imposed. This spare capacity can be filled with either positive sources (PL and SL) or rather negative sources (EL). Through reflection, positive load is activated and the freed‐up state transitions to the *effortful* state. In this state, cognitive resources are reallocated more efficiently to maintain control over performance.

Reflection is a form of cognitive restructuring vital in every practice of medicine.^58^ By interconnecting working memory and long‐term memory, it allows for creative solutions or a ‘fresh look’ by redefining the given problem. Reflection may improve learning as well as performance,^59^ since the act of cognitive restructuring promotes the development of mental models. Diverse medical training has included reflective activities to promote learning processes.^12^, ^60^

#### 3.2.3 Drifting

When spare capacity is devoted to EL (eg listening to the radio during surgery), the freed‐up state transitions to rather negative states such as drifting, where cognitive resources are not invested in essential monitoring activities. By removing the source of EL (eg turning off the radio), this state may revert to the freed‐up state and move forward to the effortful state by increasing SL.

## 4 TRAINING: PRACTICAL IMPLEMENTATION

Now that we understand the importance and processes of pausing, the next issue to be addressed how to train it. We suggest that (1) pausing should be trained within the regular medical curriculum from the start (intertwining), (2) customised support should be provided until the learner can make pauses independently (scaffolding) and (3) auxiliary tools (eg checklists) and learning environments (eg computer‐based simulation) can be used.

### 4.1 Intertwining and scaffolding

Pausing skills cannot be taught by a separate course alone since it is a domain‐general secondary skill that is interlaced with domain‐specific primary tasks.^18^ Research in education has shown that teaching secondary skills outside of primary skills training consistently fails.^61^ Moreover, transitioning between different cognitive control levels is not feasible without domain‐specific schemas in long‐term memory. Thus, it is necessary to *intertwine* pausing skills training with a primary skills training curriculum from a very early stage.

Given this necessity, the question that arises next is how to guide novices throughout the curricula. Making a pause meaningful is a highly advanced skill that requires clinical expertise and workplace experience. According to instructional design models,^18^ training programmes for complex skills should be presented in a simple‐to‐complex manner, through scaffolding where support and guidance gradually decrease as learners become more experienced. While scaffolding is generally referred to as a technique for domain‐specific skills, *second‐order scaffolding* is the technique designated for domain‐general skills.^18^

**FIGURE 3:**
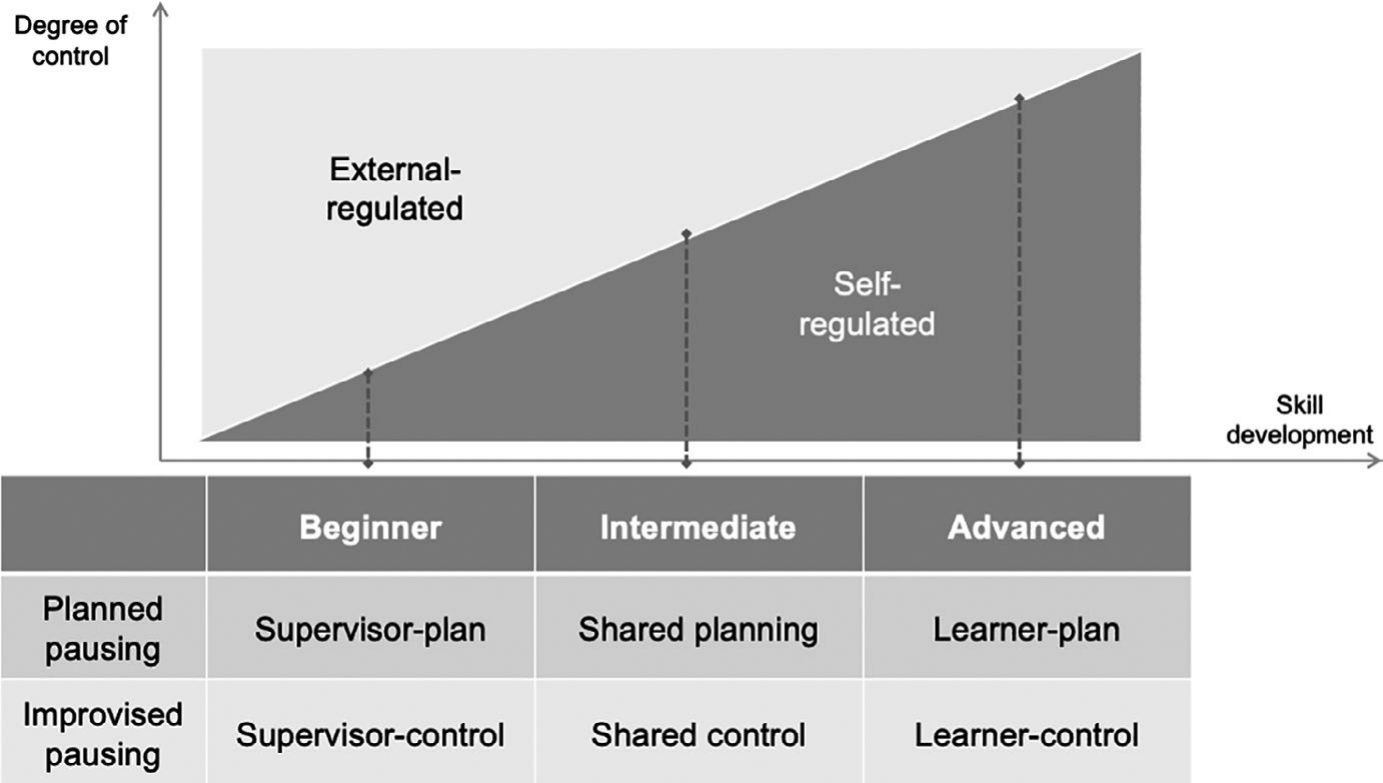
Second‐order scaffolding to improve pausing skills. While first‐order scaffolding applies to domain‐specific skills, second‐order scaffolding pertains to domain‐general skills. As a learner becomes more experienced, the supervisor's support in planning

Figure 3 demonstrates a second‐order scaffolding model for pausing skills. Since practical skills such as pausing can only be taught by hands‐on experience,^62^ some degree of direct control to initiate pauses should be granted to learners throughout a program. During this program, supervisors have a dual responsibility: they must transfer short‐term and direct control to learners while maintaining global control of the training.^63^ To find a balance, supervisors should first assess the learner's level of competence, after which both supervisor and resident can negotiate the distribution of control. The degree of supervisor control should be maximised for beginners (ie external‐regulated) and gradually reduces in favour of learner control as the learner becomes experienced (ie self‐regulated).

To teach proactive planning of pausing, learners should have the opportunity to plan for a scenario, rather than merely having a brief look at the patient chart. During this activity, supervisors should provide a framework to predict the landmines that require special measures. By explicitly locating the required focus, learners can deploy either informal or formal timeouts. To maximise learning opportunities, this plan should be thorough and specific to a particular case (eg ‘for this scenario, I will pause to be extra reflective at this point’).^63^

Learners can train improvisation of pausing by modelling the initiation of in‐action pauses. In the case of novices, the supervisor should take full control of such initiation, while the learner acts as observer. By attending various unexpected real‐life events (eg manpower shortage, malfunctioning equipment), the learner can develop his or her own measures (eg ‘if this happens to me, I will quickly assess how much time I have and initiate a timeout’). As the learner becomes more experienced, the supervisor's support in planning and improvising would gradually disappear, eventually resulting in full control by the learner.

In many training programmes, prompting learners to construct such If‐Then cognitive rules^18^ as an anticipatory strategy is already a common teaching method. Thus, integrating these cognitive rules for pausing might be easily embedded in existing programmes. Additionally, using auxiliary tools such as checklists or debriefings can be a beneficial approach to scaffolding. For instance, diagnostic checklists can help learners to train what to reflect on during pauses. Debriefing about how the executed pauses contributed to performance could also afford additional learning opportunities.

### 4.2 Simulations with a pause function

Simulated task environments offer favourable learning opportunities for medical training.^18^ They present diverse scenarios without risking patient safety or loss of materials, provide better control over the format of learning content (eg sequence of tasks, support and guidance) and make it possible to pace the task progress. This flexibility in format and pace facilitates second‐order scaffolding for pausing skills: Instructors can tailor their level of intervention to teach pausing by modulating the task progress, as learners’ competency grows.

A promising way of training pausing skills is to use computer‐based simulation (CBS) which allows for easy instalment and practice of pause functions. Diverse CBS formats, such as web‐based multimedia, serious games and virtual or augmented reality, have been used to replicate clinical settings.^40^, ^64^ In any given format, learners can practice the planning and improvisation of pauses merely by pressing a pause button. Empirical studies have shown that novices experience more cognitive load during CBS training than experts do, and that pausing may significantly help them to manage this load, especially during moments of panic (eg when virtual patients have a seizure).^40^, ^65^ However, since students reportedly tend not to understand the importance of pausing,^65^ CBS training with a pause function should include specific instructions for students to acknowledge the importance to initiate effective pauses by referring to relevant cues.

Another advantage of using CBS is that it produces objective real‐time data (eg log data of serious games) about performance that can be used as these cues. By integrating it with other types of data such as eye movements, the data can become even richer. Eye‐tracking is a novel technique that provides specific information about users’ cognitive processes during human‐computer interaction.^66^ For instance, the level of self‐monitoring or vigilance can be measured by recording how gazes are allocated across different objects.^40^ The level of cognitive load can be also measured in real time via pupillometry.^41^, ^67^

## 5 DISCUSSION

In this article, we identified pausing as a professional skill used to interrupt negative momentum and facilitate reflective performance and learning. The constituents of pausing skills were categorised into two phases: the decision‐making phase (planning, improvising and self‐monitoring) and the executive phase (relaxation and reflection). We argued that training programmes should be systematically designed to cultivate pausing skills.

The main contribution of this work lies in that it connects key concepts in safety culture that have been discussed in different contexts and brings them back to the fundamental principles of the medical pause. We have linked the practical observations and strategies from studies on slowing‐down phenomena identified by Moulton et al^20^, ^23^, ^33^ and checklists by Pronovost et al^68^, ^69^ We then recognised the cognitive mechanisms shared between the basic concepts in these studies and integrated them within a broad range of clinical activity. This will move the literature one step further as it supports the arguments of the previous studies on formal or informal timeouts and allows future researchers to build upon the literature through our newly developed understanding. Moreover, since the idea of the medical pause can be easily applied to various health professions, more practitioners may consider how to adopt a simple habit of pausing, allowing the existing discussions on slowing down and checklists to develop.

Another contribution of this work is that it provides practical techniques to implement pausing in training programmes as well as examples of application, which serves as a translational education effort. This provision may be of value to educators seeking to design better training programmes that foster the habit of reflective practice. They can start by introducing simple applications such as checklists that prompt learners to plan and improvise pauses on their own. Moreover, we discussed cognitive constructs using the new terms of primary and secondary cognitive load, which may facilitate explicit communication for supervisors and learners to jointly develop second‐order scaffolding.

We identify two limitations to this work. First, we did not consider other factors that might influence the practice of pausing (eg personality, emotions and social pressure). For instance, social pressure to make enjoyable conversation during an operation would likely affect the decision making of pausing. This social aspect of pausing can be further studied in the field of social capital in medicine. Second, since we mainly focused on theoretical conceptualisation, empirical evidence is lacking. We, therefore, welcome future studies that empirically verify the effects of medical pausing on performance and cognitive load, and the pragmatic consequences of pause training.

Medical practitioners, being either overloaded or underloaded, can lose control over their performance leading to undesirable consequences. To improve patient safety by effecting cultural and fundamental changes, the acknowledgement of the medical pause should be nurtured through communication, education and skill‐building opportunities. As a result, mastering the medical pause may become an integral part of clinical expertise, in such a way that medical professionals strategically deploy pauses in the heat of the moment by knowing when and how to pause.

## CONFLICT OF INTEREST

No competing interests exist with regard to this article.

## AUTHOR CONTRIBUTIONS

Joy Yeonjoo Lee is the main author of this work. She contributed to (1) the conception or design of the work, (2) drafting and revising the manuscript critically, (3) final approval of the manuscript and (4) agreement to be accountable for all aspects of the work. Adam Szulewski is a coauthor of this work. As an expert in emergency medicine, he contributed to (1) the conception or design of the work, (2) drafting and revising the manuscript critically by giving concrete examples from his work experience, (3) final approval of the manuscript and (4) agreement to be accountable for all aspects of the work. John Q. Young is a coauthor of this work. As an expert in medical education and psychiatry, he contributed to (1) the conception or design of the work especially making sharp ideas about safety culture in medicine, (2) drafting and revising the manuscript critically by giving concrete examples from his work experience, (3) final approval of the manuscript and (4) agreement to be accountable for all aspects of the work. Jeroen Donkers is a coauthor of this work and a supervisor of Joy Yeonjoo Lee. As an expert in medical education, he contributed to (1) the conception or design of the work, (2) drafting and revising the manuscript critically by giving concrete examples from his work experience, (3) final approval of the manuscript and (4) agreement to be accountable for all aspects of the work. Halszke Jarodzka is a coauthor of this work, and a supervisor of Joy Yeonjoo Lee. As an expert in education sciences, she contributed to (1) the conception or design of the work, (2) drafting and revising the manuscript critically, (3) final approval of the manuscript and (4) agreement to be accountable for all aspects of the work. Jeroen van Merrienboer is a coauthor of this work and a supervisor of Joy Yeonjoo Lee. As an expert in theories in medical education, such as complex learning and cognitive load theory, he contributed to (1) the conception or design of the work, (2) drafting and revising the manuscript critically, (3) final approval of the manuscript and (4) agreement to be accountable for all aspects of the work.

## ETHICAL APPROVAL

Not applicable for ethical approval. This is a theoretical paper.

## References

[1] Institute of Medicine . To Err is Human: Building a Safer Health System, vol. 6. Washington, DC: National Academies Press; 2000. 25077248

[2] Bates DW , Singh H . Two decades since to err is human: an assessment of progress and emerging priorities in patient safety. Health Aff. 2018;37(11):1736‐1743. 10.1377/hlthaff.2018.0738 30395508

[3] Institute of Medicine . Crossing the Quality Chasm: A New Health System for the 21st Century. Washington, DC: The National Academies Press; 2001. 25057539

[4] Joint Commission on Accreditation of Healthcare Organizations . Joint Comprehensive Accreditation Manual: CAMH for Hospitals: The Official Handbook. Oakbrook Terrace, IL: Joint Commission on Accreditation of Healthcare Organizations; 2009.

[5] Kelly JJ , Farley H , O’Cain C , et al. A survey of the use of time‐out protocols in emergency medicine. Jt Comm J Qual Patient Saf. 2011;37(6):285‐288. 10.1016/S1553-7250(11)37036-5 21706988

[6] Haynes AB , Weiser TG , Berry WR , et al. A surgical safety checklist to reduce morbidity and mortality in a global population. N Engl J Med. 2009;360(5):491‐499. 10.1056/NEJMsa0810119 19144931

[7] Altpeter T , Luckhardt K , Lewis JN , Harken AH , Polk HC Jr . Expanded surgical time out: a key to real‐time data collection and quality improvement. J Am Coll Surg. 2007;204(4):527‐532. 10.1016/j.jamcollsurg.2007.01.009 17382210

[8] Rall M , Glavin R , Flin R . The ‘10‐seconds‐for‐10‐minutes principle’. Bull R Coll Anaesth. 2008;51:2,614‐612,616.

[9] Sparks EA , Wehbe‐Janek H , Johnson RL , Smythe WR , Papaconstantinou HT . Surgical safety checklist compliance: a job done poorly!. J Am Coll Surg. 2013;217(5):867‐873.e863. 10.1016/j.jamcollsurg.2013.07.393 23973104

[10] Rydenfalt C , Johansson G , Odenrick P , Akerman K , Larsson PA . Compliance with the WHO Surgical Safety Checklist: deviations and possible improvements. Int J Qual Health Care. 2013;25(2):182‐187. 10.1093/intqhc/mzt004 23335056

[11] Mahajan RP . The WHO surgical checklist. Best Pract Res Clin Anaesthesiol. 2011;25(2):161‐168. 10.1016/j.bpa.2011.02.002 21550541

[12] Schmutz JB , Eppich WJ . Promoting learning and patient care through shared reflection. Acad Med. 2017;92(11):1555‐1563. 10.1097/acm.0000000000001688 28445215

[13] Melnyk BM . Culture eats strategy every time: what works in building and sustaining an evidence‐based practice culture in healthcare systems. Worldviews Evid Based Nurs. 2016;13(2):99‐101. 10.1111/wvn.12161 27062247

[14] Elstein AS , Schwarz A . Clinical problem solving and diagnostic decision making: selective review of the cognitive literature. BMJ. 2002;324(7339):729‐732. 10.1136/bmj.324.7339.729 11909793 PMC1122649

[15] Evans JSBT . Dual‐processing accounts of reasoning, judgment, and social cognition. Annu Rev Psychol. 2008;59(1):255‐278. 10.1146/annurev.psych.59.103006.093629 18154502

[16] Olsen SE , Rasmussen J . The reflective expert and the prenovice: notes on skill‐, rule‐and knowledge‐based performance in the setting of instruction and training. In: Bainbridge L , Quintanilla SAR , eds. Developing Skills with Information Technology. Chichester: Wiley; 1989:9‐33.

[17] Sweller J , Van Merriënboer JJ , Paas F . Cognitive architecture and instructional design: 20 years later. Educ Psychol Rev. 2019;31(2):261‐292. 10.1007/s10648-019-09465-5

[18] Van Merriënboer JJ , Kirschner PA . Ten Steps to Complex Learning: A Systematic Approach to Four‐Component Instructional Design. New York, NY: Routledge; 2018.

[19] Van Merrienboer JJ , Sweller J . Cognitive load theory in health professional education: design principles and strategies. Med Educ. 2010;44(1):85‐93. 10.1111/j.1365-2923.2009.03498.x 20078759

[20] Moulton CA , Regehr G , Mylopoulos M , Macrae HM . Slowing down when you should: a new model of expert judgment. Acad Med. 2007;82(Suppl):S109‐S116. 10.1097/acm.0b013e3181405a76 17895673

[21] Dreyfus HL , Dreyfus SE . Mind Over Machine. New York, NY: The Free Press; 1986.

[22] Ericsson KA . Deliberate practice and the acquisition and maintenance of expert performance in medicine and related domains. Acad Med. 2004;79(10):S70‐S81. 10.1097/00001888-200410001-00022 15383395

[23] Moulton CA , Epstein RM . Self‐monitoring in surgical practice: slowing down when you should. In: Fry H , Kneebone R , eds. Surgical Education. Dordrecht: Springer; 2011:169‐182.

[24] Moulton CA , Regehr G , Lingard L , Merritt C , MacRae H . Slowing down to stay out of trouble in the operating room: remaining attentive in automaticity. Acad Med. 2010;85(10):1571‐1577. 10.1097/ACM.0b013e3181f073dd 20881677

[25] Briki W , Markman KD , Coudevylle G , Sinnapah S , Hue O . Momentum sequence and environmental climate influence levels of perceived psychological momentum within a sport competition. Eur J Sport Sci. 2016;16(3):350‐357. 10.1080/17461391.2015.1062566 26263831

[26] Deemer ED , Derosa PA , Duhon SA , Dotterer AM . Psychological momentum and inertia: toward a model of academic motivation. J Career Dev. 2019. 10.1177/0894845319848847

[27] McElhatton J , Drew C . Time pressure as a causal factor in aviation safety incidents‐ The 'hurry‐up' syndrome. Paper presented at: International Symposium on Aviation Psychology, 7 th, Columbus, OH; 1993.

[28] Smallwood J , Mrazek MD , Schooler JW . Medicine for the wandering mind: mind wandering in medical practice. Med Educ. 2011;45(11):1072‐1080. 10.1111/j.1365-2923.2011.04074.x 21988623

[29] Ericsson KA . The influence of experience and deliberate practice on the development of superior expert performance. In: Ericsson K , Charness N , Feltovich P , Hoffman R , eds. The Cambridge Handbook of Expertise and Expert Performance. Cambridge: Cambridge University Press; 2006;38, 683‐704. 10.1017/CBO9780511816796.038.

[30] Wallace JE , Lemaire JB , Ghali WA . Physician wellness: a missing quality indicator. Lancet. 2009;374(9702):1714‐1721. 10.1016/s0140-6736(09)61424-0 19914516

[31] Beilock SL , Bertenthal BI , Hoerger M , Carr TH . When does haste make waste? Speed‐accuracy tradeoff, skill level, and the tools of the trade. J Exp Psychol Appl. 2008;14(4):340. 19102617 10.1037/a0012859

[32] Heitz RP . The speed‐accuracy tradeoff: history, physiology, methodology, and behavior. Front Neurosci. 2014;8(150):1–19. 10.3389/fnins.2014.00150 24966810 PMC4052662

[33] Moulton CA , Regehr G , Lingard L , Merritt C , Macrae H . ‘Slowing down when you should’: initiators and influences of the transition from the routine to the effortful. J Gastrointest Surg. 2010;14(6):1019‐1026. 10.1007/s11605-010-1178-y 20309647

[34] Al S , Tariq M , Alawar B , Al N . Vigilance decrement and enhancement techniques: a review. Brain Sci. 2019;9(8):178. 10.3390/brainsci9080178 PMC6721323 31357524

[35] De Bruin ABH , Van Merriënboer JJG . Bridging cognitive load and self‐regulated learning research: a complementary approach to contemporary issues in educational research. Learn Instruct. 2017;51:1‐9. 10.1016/j.learninstruc.2017.06.001

[36] Young JQ , Van Merrienboer J , Durning S , Ten Cate O . Cognitive load theory: implications for medical education: AMEE Guide No. 86. Med Teach. 2014;36(5):371–384. 10.3109/0142159X.2014.889290 24593808

[37] Warm JS , Parasuraman R , Matthews G . Vigilance requires hard mental work and is stressful. Hum Factors. 2008;50(3):433‐441. 10.1518/001872008x312152 18689050

[38] Lingard L , Haber RJ . Teaching and learning communication in medicine: a rhetorical approach. Acad Med. 1999;74(5):507‐510. 10.1097/00001888-199905000-00015 10353281

[39] Blissett S , Sibbald M , Kok E , van Merrienboer J . Optimizing self‐regulation of performance: is mental effort a cue? Adv Health Sci Educ. 2018;23(5):891‐898. 10.1007/s10459-018-9838-x 29948414

[40] Lee JY , Donkers J , Jarodzka H , van Merriënboer JJ . How prior knowledge affects problem‐solving performance in a medical simulation game: using game‐logs and eye‐tracking. Comput Human Behav. 2019;99:268‐277. 10.1016/j.chb.2019.05.035

[41] Szulewski A , Roth N , Howes D . The use of task‐evoked pupillary response as an objective measure of cognitive load in novices and trained physicians: a new tool for the assessment of expertise. Acad Med. 2015;90(7):981‐987. 10.1097/Acm.0000000000000677 25738386

[42] Young JQ , Van Dijk SM , O'Sullivan PS , Custers EJ , Irby DM , Ten Cate O . Influence of learner knowledge and case complexity on handover accuracy and cognitive load: results from a simulation study. Med Educ. 2016;50(9):969‐978. 10.1111/medu.13107 27562896

[43] Shear T , Deshur M , Avram MJ , et al. Procedural timeout compliance is improved with real‐time clinical decision support. J Patient Saf. 2018;14(3):148‐152. 10.1097/pts.0000000000000185 25894382

[44] Ariga A , Lleras A . Brief and rare mental “breaks” keep you focused: deactivation and reactivation of task goals preempt vigilance decrements. Cognition. 2011;118(3):439‐443. 10.1016/j.cognition.2010.12.007 21211793

[45] Rosenbluth G , Destino LA , Starmer AJ , et al. I‐PASS Handoff Program: use of a campaign to effect transformational change. Pediatr Qual Saf. 2018;3(4):e088. 30229199 10.1097/pq9.0000000000000088 PMC6135553

[46] Shahid S , Situation TS . Situation, Background, Assessment, Recommendation (SBAR) communication tool for handoff in health care – a narrative review. Saf Health. 2018;4(7): 1–9. 10.1186/s40886-018-0073-1

[47] LeBlanc VR . The effects of acute stress on performance: implications for health professions education. Acad Med. 2009;84(10):S25‐S33. 19907380 10.1097/ACM.0b013e3181b37b8f

[48] Yerkes RM , Dodson JD . The relation of strength of stimulus to rapidity of habit‐formation. J Comp Neurol Psychol. 1908;18(5):459‐482. 10.1002/cne.920180503

[49] McBee E , Ratcliffe T , Picho K , et al. Contextual factors and clinical reasoning: differences in diagnostic and therapeutic reasoning in board certified versus resident physicians. BMC Med Educ. 2017;17(1):211. 29141616 10.1186/s12909-017-1041-x PMC5688653

[50] Szulewski A , Howes D , van Merriënboer JJG , Sweller Jo . From theory to practice: the application of cognitive load theory to the practice of medicine. Acad Med. 2021;96(1):24‐30. 10.1097/ACM.0000000000003524 32496287

[51] Chen O , Castro‐Alonso JC , Paas F , Sweller J . Extending cognitive load theory to incorporate working memory resource depletion: evidence from the spacing effect. Educ Psychol Rev. 2018;30(2):483‐501. 10.1007/s10648-017-9426-2

[52] Parnabas VA , Mahamood Y , Parnabas J , Abdullah NM . The relationship between relaxation techniques and sport performance. Univ J Psychol. 2014;2(3):108‐112. 10.13189/ujp.2014.020302

[53] Young JQ , Wachter RM , ten Cate O , O'Sullivan PS , Irby DM . Advancing the next generation of handover research and practice with cognitive load theory. BMJ Qual Saf. 2016;25(2):66‐70. 10.1136/bmjqs-2015-004181 26786521

[54] Paul G , Barb Elam J , Verhulst S . A longitudinal study of students' perceptions of using deep breathing meditation to reduce testing stresses. Teach Learn Med. 2007;19(3):287‐292. 10.1080/10401330701366754 17594225

[55] Perciavalle V , Blandini M , Fecarotta P , et al. The role of deep breathing on stress. Neurol Sci. 2017;38(3):451‐458. 10.1007/s10072-016-2790-8 27995346

[56] Khng KH . A better state‐of‐mind: deep breathing reduces state anxiety and enhances test performance through regulating test cognitions in children. Cogn Emot. 2017;31(7):1502‐1510. 10.1080/02699931.2016.1233095 27666392

[57] Yadav G , Mutha PK . Deep breathing practice facilitates retention of newly learned motor skills. Sci Rep. 2016;6(1):37069. 10.1038/srep37069 27841345 PMC5107920

[58] Sandars J . The use of reflection in medical education: AMEE Guide No. 44. Med Teach. 2009;31(8):685‐695. 10.1080/01421590903050374 19811204

[59] Ohtani K , Hisasaka T . Beyond intelligence: a meta‐analytic review of the relationship among metacognition, intelligence, and academic performance. Metacogn Learn. 2018;13(2):179‐212. 10.1007/s11409-018-9183-8

[60] Branch WT Jr . Small‐group teaching emphasizing reflection can positively influence medical students' values. Acad Med. 2001;76(12):1171‐1172. 11739035 10.1097/00001888-200112000-00001

[61] Tricot A , Sweller J . Domain‐specific knowledge and why teaching generic skills does not work. Educ Psychol Rev. 2014;26(2):265‐283. 10.1007/s10648-013-9243-1

[62] Schön DA . Educating the reflective practitioner: toward a new design for teaching and learning in the professions. Austral J Adult Learn. 2010;50(2):448‐451.

[63] St‐Martin L , Patel P , Gallinger J , Moulton C‐A . Teaching the slowing‐down moments of operative judgment. Surg Clin North Am. 2012;92(1):125‐135. 10.1016/j.suc.2011.12.001 22269266

[64] Pantelidis P , Chorti A , Papagiouvanni I , et al. Virtual and augmented reality in medical education. In: Tsoulfas G , ed. Medical and Surgical Education: Past, Present and Future. London, UK: IntechOpen; 2018:77‐97.

[65] Lee JY , Donkers J , Jarodzka H , Sellenraad G , Van Merriënboer JJG . Different effects of pausing on cognitive load in a medical simulation game. Comput Human Behav. 2020;110:106385. 10.1016/j.chb.2020.106385

[66] Holmqvist K , Andersson R . Eye‐Tracking: A Comprehensive Guide to Methods, Paradigms and Measures. Lund, Sweden: Lund Eye‐Tracking Research Institute; 2017.

[67] Szulewski A , Gegenfurtner A , Howes DW , Sivilotti ML , van Merriënboer JJ . Measuring physician cognitive load: validity evidence for a physiologic and a psychometric tool. Adv Health Sci Educ. 2017;22(4):951‐968. 10.1007/s10459-016-9725-2 27787677

[68] Hales BM , Pronovost PJ . The checklist—a tool for error management and performance improvement. J Crit Care. 2006;21(3):231‐235. 10.1016/j.jcrc.2006.06.002 16990087

[69] Pronovost PJ , Bo‐Linn GW . Preventing patient harms through systems of care. JAMA. 2012;308(8):769. 10.1001/jama.2012.9537 22910751

